# Nontraumatic Myositis Ossificans of Hip: A Case Presentation

**DOI:** 10.1155/2016/1982656

**Published:** 2016-06-29

**Authors:** Yunus Oc, Muhammed Sefa Ozcan, Hasan Basri Sezer, Bekir Eray Kilinc, Osman Tugrul Eren

**Affiliations:** ^1^Sisli Hamidiye Etfal Training and Research Hospital, 19 Mayıs Mahallesi, Sisli, 34360 Istanbul, Turkey; ^2^Igdir State Hospital Orthopaedics and Traumatology Department, Igdir, Turkey

## Abstract

In most of the cases trauma is the leading etiology and the nontraumatic myositis ossificans (MO) is a very rare condition. We present an MO case without any trauma occurring. A 36-year-old female patient with a history of pain and restriction of range of motion of the left hip was admitted. Hip motions were restricted with 10–60° of flexion, 10° of internal rotation, 20° of external rotation, 10° of abduction, and 10° of adduction. There was no history of trauma and familial involvement. The biopsy of the lesion revealed mature bone tissue confirming our diagnosis of MO. The mass was removed surgically and postoperatively the patient was treated with a single dose radiotherapy with 800 gyc. MO is a benign and well differentiated bone formation or in other words heterotopic ossification of the muscle tissue. It has a prevalence of less than 1/1 million. Trauma is the most frequent etiological factor seen in almost 60–75% of the cases. Nontraumatic MO is very rare in the literature. Our patient had no history of trauma or familial involvement. Combination of the surgical excision with radiotherapy in the treatment of the MO of the hip may give satisfactory results.

## 1. Introduction

Myositis ossificans (MO) is a nonneoplastic and benign condition in which there is an increased activity of periarticular tissues resulting in intramuscular bone formation [1]. In most of the cases trauma is the leading etiology and the nontraumatic MO is a very rare condition. It may affect any localization in the human body but selectively the areas which are susceptible to the trauma are involved such as hip, elbow, and wrist [2]. Biopsy may be required in some cases for differential diagnosis. This paper presents a very rare case of nontraumatic heterotopic ossification of hip.

## 2. Presentation of Case

A 36-year-old female patient with a history of the pain and the restriction of range of motion of the left hip admitted to our outpatient clinic. She complained of a mass in the left hip diagnosed by an orthopaedic surgeon 2 years ago and she was followed up with only clinical observation. Hip motions were restricted with a 10°–60° of flexion, 10° of internal rotation, 20° of external rotation, 10° of abduction, and 10° of adduction. There was no history of trauma or familial involvement.

**Figure 1:**
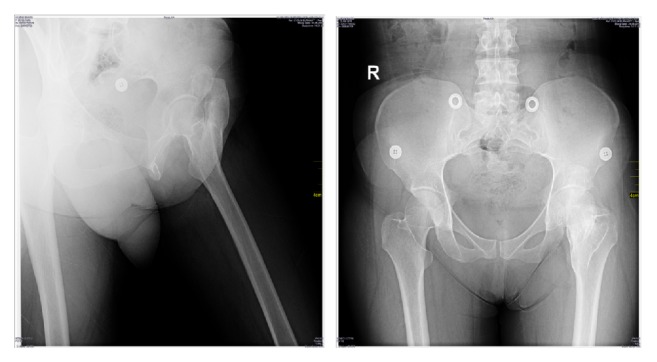
Preoperative radiographic evaluation with X-ray.

Radiographic evaluation of the patient with X-ray revealed a 13 × 6 cm radiopaque mass extending from the anterior border of acetabulum to the trochanter minor medially and the trochanter major laterally (Figure 1).

**Figure 2:**
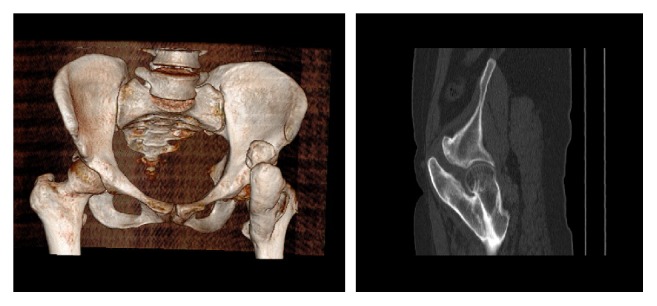
Preoperative radiographic evaluation with 3D and sagittal view of CT.

**Figure 3:**
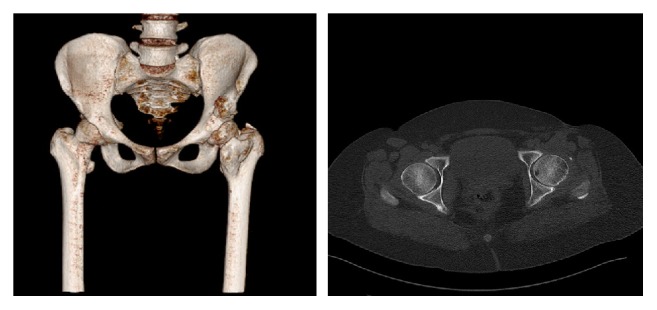
Post-op 2nd-year radiographic evaluation with 3D and axial view of CT.

The CT revealed a mass which was bridging from anterior aspect of coxofemoral joint to the trochanter minor with a large attachment (Figure 2). CT was applied at 2-year follow-up to show the removal of the mass (Figure 3).

**Figure 4:**
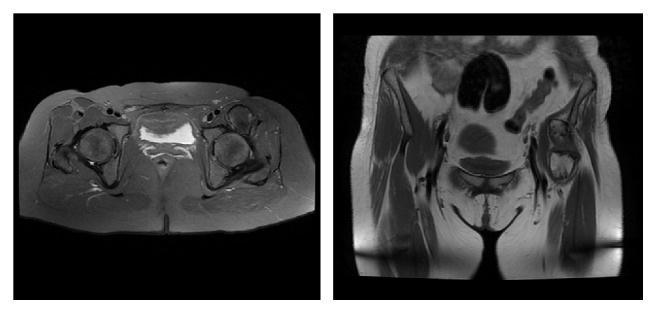
Preoperative radiographic evaluation MRI.

The MRI revealed a mass which was broader in the intertrochanteric line where it was in a close relation to the bone. It was 11 cm long and 3 cm wide at the broadest part (Figure 4). The mass was osseous in character which was located along the lateral border of the iliopsoas muscle in its craniocaudal extension. Intraoperatively, the mass was seen starting from the superoanterior edge of the acetabulum with a small portion in the rectus femoris muscle and ending with a large attachment part to the anterior aspect of femur on the trochanter minor level. Furthermore, it was building an osseous bridge from anterior aspect of coxofemoral joint that was limiting the range of motion of the hip. We realized that after removal of the mass the range of motion of the hip was totally released.

There was another mass sized 35 × 19 mm located superiorly close to the iliac bone anterior to the acetabulum. The soft tissue between 2 masses was edematous and inflammatory in character resembling the MO.

**Figure 5:**
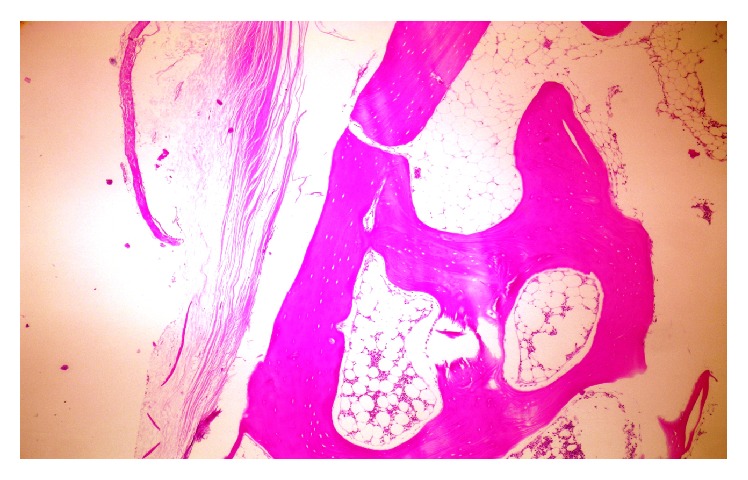
Histological examination of the specimen showing mature osteoid under 40x magnification.

**Figure 6:**
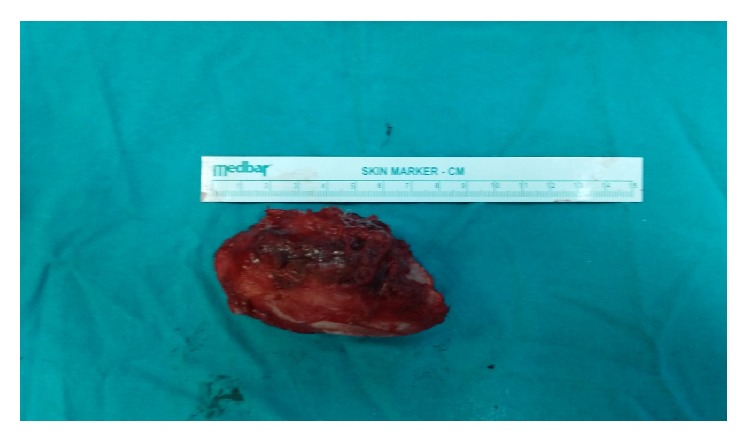
Surgically excised mass.

The biopsy of the lesion revealed mature bone tissue confirming our diagnosis of MO (Figure 5). The mass was removed surgically (Figure 6) and postoperatively the patient was treated with a single dose radiotherapy with 800 gyc.

There was a dramatic decrease in the pain and increase in the range of motion postoperatively. In the last follow-up examination the hip was able to reach 120° of flexion, 10° of extension, 30° of internal rotation, 40° of external rotation, 40° of abduction, and 30° of adduction.

## 3. Discussion

Myositis ossificans is a benign and well differentiated bone formation or in other words heterotopic ossification of the muscle tissue [1]. It has a prevalence of less than 1/1 million. There is no sexual predominance [3]. Trauma is the most frequent etiological factor seen in almost 60–75% of the cases [4–8]. It is believed that after a distinguishable trauma there occurs a tissue necrosis or bleeding initiating an uncontrolled vascular and fibroblastic activity resulting with bone formation [7]. Although unproven, some other etiological mechanisms were also hypothesized. One of the theories claims osteoblasts that are freed from periost and trapped in the soft tissues as the provocateur of the MO [6]. The other mechanism is the “ectopic calcification islands” theory which accuses periosteal tissue itself to be displaced into the soft tissues because of the impact of the trauma causing MO [9]. Tabes dorsalis, syringomyelia, poliomyelitis, paraplegia, tetanus, and hemophilia may play a role as the underlying pathology [9–11]. In the presence of such conditions MO may occur even; passive range of motion exercises is carried out. Burns, infections, and drug abuse are other rare conditions which may cause MO [9, 10].

Nontraumatic MO is very rare in the literature [7, 8, 12, 13]. Repetitive microtrauma, tissue ischemia, and inflammation were addressed as the causal mechanisms of the nontraumatic MO [7, 12]. MO of the hip occurs more frequently in patients experiencing palsy, subdural or epidural bleeding, and hip operation. Our case is free of all of those conditions. Fibrodysplasia ossificans progressive is another disease which presents with nontraumatic MO. It is a rare disease of 5- to 25-year-old population expressing autosomal dominant inheritance [9]. Clinically it is a progressive disease and may present with thumb and toe anomalies [3, 9]. In our case there was no family history or concomitant hand or toe anomalies.

To our knowledge our case is unique of being nontraumatic and having no simultaneous predisposing factors.

The pattern of progression in MO is pathognomonic by expressing a peripheral to central manner [3, 7, 10, 13]. Histologically collagen producing cells are located in the center and increased osteoblastic activity and immature bone lies in the intermediate zone and lamellar bone in the peripherally [13].

Clinically there is a formation of a painful mass at the region of trauma within 7–10 days [4]. Between 10 days and 6 weeks there appear to be irregular osseous fragments in this mass [4, 6, 8]. Cortical bone production takes place between 6 and 8 weeks [10]. From 10 weeks to 6 months the typical egg shell appearance of central zone is visible [4, 10]. Maturation of the mass takes place between 6 and 8 months and the mass may shrink to some degree [4, 6, 8]. Some lesions decrease in volume and some disappear within 1-2 years [4, 6].

MRI findings demonstrate heterogeneity due to the histological structure of the MO lesions [8, 10]. In the early period of the disease in T2 MRI section there is a dark and nonhomogenous intensity distribution in the central zone [8, 10]. The emergence of a hyperintensive ring around a hypointensive core is the sign of maturation of the mass [8, 10, 11]. There is no specific radiological finding of the nontraumatic MO.

MO is generally self-limited pathology [10]. There is a possibility of the spontaneous regression; thus surgical excision is not the primary choice of treatment by most of the surgeons [3]. Typical lesions may be followed with clinical and radiological observation [10]. Surgical indications include pain, increasing diameter of the mass, deteriorating local tendon or muscle function, and decreasing functional ability of the patient [10, 13, 14]. Such lesions may be excised after maturation.

Radiotherapy (RT) may decrease the diameter of the mass and may increase the maturation of the mass [14]. In the treatment of MO, one low dose RT was performed in many cases and it was seen very effective. Gokkus et al. reported that 24 hours after operation one low dose RT was effective in their case [15]. In another case report, Pakos et al. showed that RT treatment with combined indomethacin protocol was an effective treatment in MO [16]. Our case had a 3-year history with no regression and progressive deterioration of the left hip function. Our diagnosis was confirmed with the biopsy of the lesion. After excision of the mass one dose of radiotherapy (800 gyc) was administered. Postoperatively, there was a dramatic decrease in the pain and the patient had closely normal hip range of motion. There was no recurrence at 2-year follow-up proved by CT scan.

## 4. Conclusion

Nontraumatic MO is very rare and our case is the first case in the literature with no trauma or predisposing factors. Biopsy may be required to verify diagnosis. Combination of the surgical excision with radiotherapy in the treatment of the MO of the hip may give satisfactory results.
